# Genome-Wide Association Study Uncovers Candidate Genes Governing Oil Quality Traits in Sunflower (*Helianthus annuus* L.)

**DOI:** 10.3390/plants15070999

**Published:** 2026-03-25

**Authors:** Haifeng Yu, Yingnan Mu, Xuerui Wu, Zhibiao He, Chunling Zhang, Yang Wu, Ying Sun, Liuxi Yi, Jun Li, Gang Wang, Jiayao Sun, Wenyu Han, Yongsheng Chen

**Affiliations:** 1 Inner Mongolia Academy of Agricultural & Animal Husbandry Sciences, Hohhot 010031, China; nkyyhf@163.com (H.Y.);; 2 Bayan Nur Institute of Agriculture and Animal Husbandry Science, Bayan Nur 015000, China; 3 Tongliao Academy of Agricultural and Animal Husbandry Sciences, Tongliao 028015, China; 4 Agricultural College, Inner Mongolia Agricultural University, Hohhot 010019, China

**Keywords:** genome-wide association study (GWAS), fatty acid, linoleic acid, single-nucleotide polymorphism (SNP), candidate gene

## Abstract

Sunflower is a globally important oilseed crop. Improving its fatty acid composition is crucial for enhancing oil quality and nutritional value. To dissect the genetic basis of quality traits, we performed genome resequencing on 203 sunflower inbred lines and conducted a genome-wide association study (GWAS) for five traits—oil content, stearic acid, palmitic acid, oleic acid, and linoleic acid—across three environments. We identified 103 significant single-nucleotide polymorphisms (SNPs) and 154 candidate genes. Notably, several associated loci were co-localized for multiple traits, suggesting pleiotropic effects or close genetic linkages. Integration with transcriptome data from developing seeds revealed that 66 candidate genes were expressed within 30 days after pollination, of which 12 showed significant differential expression between high- and low-oleic acid varieties. Functional characterization of a selected candidate, the *ω-6 fatty acid desaturase* gene (*LOC110938218*, designated *HaDES8.11*), demonstrated that the HaDES8.11-eGFP fusion protein localizes to the endoplasmic reticulum. Heterologous expression of *HaDES8.11* in *Arabidopsis thaliana* significantly increased seed linoleic acid content while decreasing oleic acid content, confirming its role in fatty acid desaturation. Our study provides novel genetic insights and valuable candidate genes for the molecular breeding of sunflower with optimized oil quality.

## 1. Introduction

Sunflower (*Helianthus annuus* L.) is a globally important oilseed crop, cultivated on over 27 million hectares with an annual production of approximately 50 million tons, playing a critical role in the world’s edible oil supply [1]. As the fourth-largest oilseed crop, sunflower oil is highly valued for its unique fatty acid composition, with unsaturated fatty acids—primarily oleic acid (18:1) and linoleic acid (18:2)—accounting for over 90% of the total fatty acids [2]. The quality and nutritional value of sunflower oil depend largely on the proportions of these fatty acids. Oleic acid, a monounsaturated fatty acid, has been associated with cardiovascular health benefits [3], enhanced oxidative stability [4], and reduced formation of trans-fatty acids during high-temperature processing [5]. Conversely, high levels of polyunsaturated linoleic acid can accelerate oil deterioration and compromise oxidative stability during storage [6].

Increasing oleic acid content while appropriately reducing linoleic acid has therefore become a major breeding objective for sunflower oil quality improvement [7,8]. High-oleic sunflower oil commands greater economic value in food processing and culinary applications due to its superior stability and health benefits. However, currently cultivated sunflower varieties predominantly feature conventional oleic acid levels, and the scarcity of high-quality specialized varieties hinders industrial upgrading. Elucidating the genetic basis of fatty acid composition and identifying key regulatory genes is therefore of great significance for accelerating the breeding of high-quality sunflower varieties.

Genome-wide association studies (GWAS) have played an indispensable role in dissecting the genetic architecture of complex quality traits in crops, providing crucial theoretical foundations and genetic resources for quality improvement [9,10]. By employing high-density SNP markers for genome-wide scanning of association panels, GWAS correlates genotypic data with phenotypic data to identify molecular markers or candidate genes tightly linked to target traits. Compared to traditional biparental linkage mapping, GWAS offers significant advantages, including no requirement for segregating populations, shorter research cycles, and higher mapping resolution [11].

In rapeseed (*Brassica napus*), GWAS studies on fatty acid composition have successfully identified associated genetic loci and candidate genes *BnaA09.PYRD*, *BnaA08.PSK1*, *BnaA08.SWI3*, *BnaC02.LTP15* and *BnaFAX1-1* [12,13,14]. For instance, Hu et al. [15] analyzed protein and seed oil content in 183 rapeseed accessions across two environments, combining 60K SNP array data for GWAS and transcriptome analysis, identifying 256 common genes with 25 likely involved in drought stress response affecting protein and fat accumulation. Gajardo et al. [16] evaluated six quality traits in 89 winter rapeseed accessions over two seasons and locations, performing GWAS with 4025 SNP markers from a 6K Illumina array, and identified 17 and 5 SNPs significantly associated with seed glucosinolate and hemicellulose content, respectively. Gubaev et al. [17] genotyping-by-sequencing (GBS) to construct a high-density SNP-based genetic map in sunflower, revealing that while a single major-effect QTL governs high oleic acid content, its phenotypic expression—whether dominant or recessive—varies significantly across genetic backgrounds. Furthermore, a potential chromosomal translocation between linkage groups 7 and 14 in one cross suggests structural variation may modulate QTL penetrance, underscoring the nuanced interplay between genotype and genetic context in molecular breeding strategies. In sunflower, GWAS has been successfully applied to identify loci associated with fatty acid composition. However, integrated multi-environment GWAS focusing on quality traits remains relatively limited, and the regulatory networks governing fatty acid biosynthesis await further elucidation.

In this study, we utilized 203 sunflower inbred lines as materials. Through phenotypic evaluation of five quality-related traits across three environments and subsequent GWAS, we aimed to mine significantly associated SNP loci and candidate genes related to sunflower fatty acid metabolism and conduct functional validation. Our findings are expected to enhance the understanding of the genetic basis and breeding potential of fatty acid traits in sunflower.

## 2. Results

### 2.1. Analysis of Quality-Related Traits in Sunflower

Measurements of five quality-related traits in 203 sunflower inbred lines (Table S1) revealed that the average coefficient of variation (CV) across three environments ranged from 1.57% to 28.47% (Table 1). Stearic acid exhibited the highest average CV (28.47%), while linoleic acid showed the lowest (1.57%). Broad-sense heritability (H^2^) ranged from 48.25% to 70.78%. Oil content had the highest H^2^ (70.78%), followed by stearic acid, palmitic acid, oleic acid, and linoleic acid (48.25%). Across the three environments, the highest mean contents for stearic acid (4.95%), linoleic acid (65.93%), and palmitic acid (6.36%) were observed at the NKY site, whereas the highest mean oleic acid (23.47%) and oil content (38.62%) were recorded at the QGT site.

**Figure 1:**
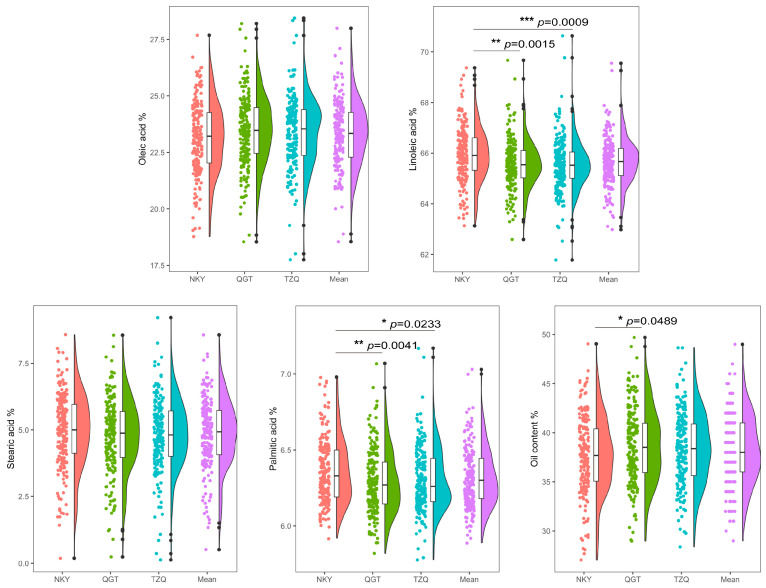
Density distribution of five quality-related traits of 203 sunflower inbred lines grown under different environments. Mean, overall mean across every environment. In the violin plot, the center line indicates the median, and the edges of the violin represent the 25th and 75th percentiles. (*, **, *** indicate *p* < 0.05, 0.01, 0.001, respectively).

**Figure 2:**
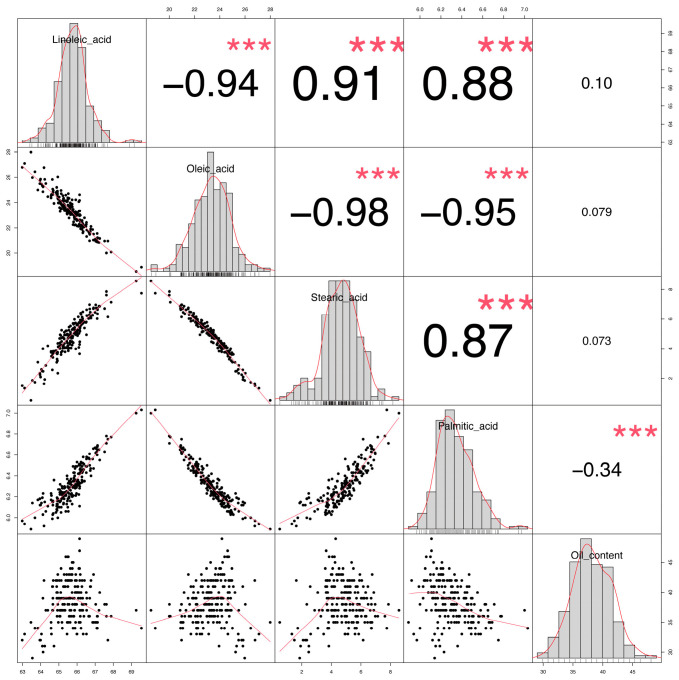
Phenotypic distribution and correlation of fatty acid contents in 203 sunflower inbred lines accessions across three environments. Statistical significance of coefficients labeled as the *p*-values. (*** indicates *p* < 0.001).

Further one-way analysis of variance (ANOVA) for the five quality traits revealed that, except for oleic acid and stearic acid contents (Figure 1), all other traits showed significant differences across the three environments. Significant differences were also observed for linoleic acid and palmitic acid contents among the different locations, while oil content differed significantly only between the NKY and QGT environments. Table S2 presents the two-way ANOVA results, revealing significant effects (*p* < 0.001) of genotype, environment, and genotype × environment interactions on each trait. Pearson correlation analysis revealed complex interrelationships among the traits (Figure 2). Oleic acid content exhibited a highly significant negative correlation with linoleic acid (r = −0.89, *p* < 0.001), stearic acid (r = −0.42, *p* < 0.01), and palmitic acid (r = −0.38, *p* < 0.01) contents. Conversely, linoleic acid content was significantly positively correlated with both stearic acid (r = 0.45, *p* < 0.001) and palmitic acid (r = 0.41, *p* < 0.001) contents. Oil content showed a highly significant negative correlation with palmitic acid (r = −0.35, *p* < 0.001) and non-significant positive correlations with oleic acid, linoleic acid, and stearic acid. These correlation patterns suggest potential genetic linkages or pleiotripic effects, indicating that individual quality traits in sunflower do not vary independently and that altering one trait may significantly influence others. Frequency distributions for all five traits approximated normality, indicating their quantitative nature.

### 2.2. SNP Marker Distributions

**Figure 3:**
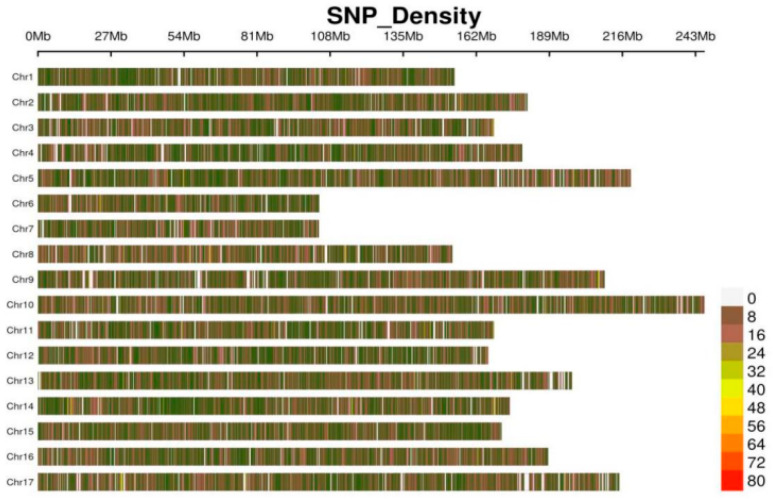
Density plot of SNPs across 17 chromosomes (Chr) in sunflower genome.

Genome resequencing of the 203 sunflower accessions yielded 19.8 Tb of data with an average sequencing depth of 20×. The average Q30 was 94.87% (range: 91.04–96.44%), and the average GC content was 44.43% (range: 41.89–46.73%). The read mapping rate and properly paired rate were 97.89% and 91.56%, respectively. Using SAMTOOLS [18] against the sunflower reference genome (GeneBank accession NO. GCA_002127325.2), 32,480,291 raw SNPs were obtained. Filtering based on a Bayesian model (depth ≥ 4, missing rate < 0.2, minor allele frequency (MAF) > 0.01) [19] resulted in 1,334,041 high-quality SNPs. On average, 13,092.81 SNPs were obtained per sample, with an average density of 2.30 SNPs per kilobase distributed across all 17 chromosomes. Chromosome 10 harbored the highest number of SNPs (98,123), while chromosome 14 had the fewest (51,332) (Figure 3).

### 2.3. Population Structure and Linkage Disequilibrium Analysis

**Figure 4:**
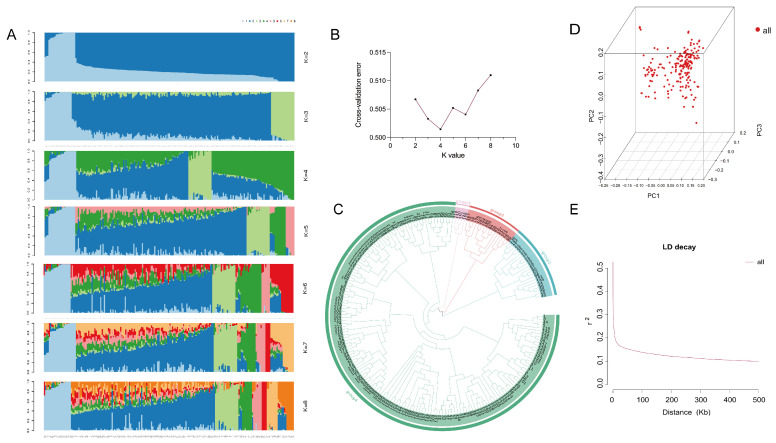
Population structure and linkage disequilibrium analysis of 203 sunflower inbred lines. (**A**) Cross-validation error for different K values (K = 2–8). (**B**) Population structure bar plot at K = 4. (**C**) Phylogenetic tree. (**D**) Principal component analysis (PCA) plot. (**E**) Genome-wide LD decay plot.

Population genetic structure and lineage information were analyzed using PLINK (v1.9) and ADMIXTURE (v1.23) software. The population structure for the 203 accessions was evaluated by setting the number of clusters (K) from 2 to 8. Based on the smallest cross-validation error (ΔK), the optimal K value was determined to be 4, dividing the panel into four subpopulations (Figure 4A, B). This clustering was corroborated by phylogenetic tree analysis (Figure 4C). Principal component analysis (PCA) showed no distinct geographical subpopulations, with accessions from different origins intermixed (Figure 4D), consistent with the phylogenetic clustering. Linkage disequilibrium (LD) decay across the genome was assessed using PopLDdecay (v3.40) by calculating the squared correlation coefficient (r^2^) for alleles of the filtered 1,334,041 SNPs (Figure 4E). The LD decay distance, defined as the physical distance at which r^2^ decays to half its maximum value (r^2^ = 0.215), was approximately 20 kb. This LD decay distance was subsequently used for defining candidate gene regions in the GWAS.

### 2.4. Identification of Quality-Related SNPs via Genome-Wide Association Study in Sunflower

**Figure 5:**
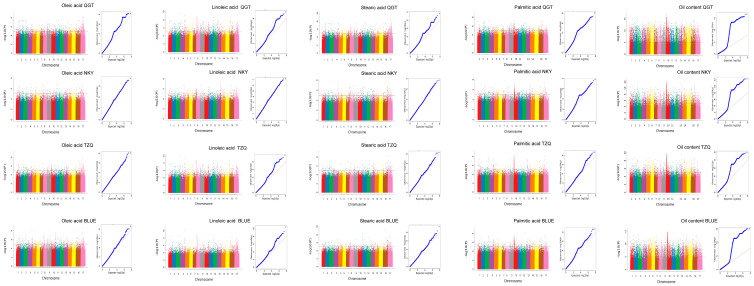
GWAS and Q-Q plot of sunflower fatty acid-related traits in three environments and the best linear unbiased Estimator (BLUE). Gray horizontal dotted line indicates significance threshold line. (−log_10_(*p*) = 5).

GWAS was performed for the five quality-related traits (oil content, linoleic acid, oleic acid, stearic acid, and palmitic acid) using phenotypic data from three individual environments and the best linear unbiased estimates (BLUE). A total of 103 significant SNP loci were identified (Figure 5, Table 2 and Table S3). Specifically, 15 SNPs were associated with oleic acid content, distributed on chromosomes 1, 2, 3, 8, 10, 11, 16, and 17, with phenotypic variation explained (PVE) ranging from 7.13% to 13.24%. Thirty SNPs were associated with linoleic acid content, located on chromosomes 1, 2, 3, 4, 5, 6, 7, 8, 11, 14, 16, and 17 (PVE: 5.39–12.67%). Twenty-one SNPs were associated with palmitic acid content, mainly on chromosomes 1, 2, 3, 4, 9, 10, 13, 16, and 17 (PVE: 5.12–10.57%). Another 21 SNPs were associated with stearic acid content, primarily on chromosomes 2, 3, 4, 8, 9, 11, 12, 16, and 17 (PVE: 5.67–11.27%). Finally, 16 SNPs were associated with oil content, distributed on chromosomes 4, 5, 6, 9, 10, 12, 16, and 17 (PVE: 6.15–14.27%).

### 2.5. Candidate Gene Identification and Expression Analysis

Candidate genes were predicted within a 20 kb region upstream and downstream of each significant SNP. A total of 154 candidate genes were identified in proximity to the 103 SNPs (Table 2 and Table S3). These genes are involved in diverse biological processes such as lipid metabolism, signal transduction, transcriptional regulation, and transport. For example, *LOC110937516* (*KCS5*, *3-ketoacyl-CoA synthase 5*) and *LOC110938218* (*omega-6 fatty acid desaturase*) are known to participate in fatty acid synthesis or modification. Notably, several SNPs were associated with multiple traits. For instance, the SNP at 104,606,151 bp on chromosome 2 was simultaneously associated with stearic acid, linoleic acid, and oleic acid, with nearby candidate genes including *LAC13* (*laccase-13*). Conversely, some SNPs were trait-specific, such as the SNP at 146,673,044 bp on chromosome 10, associated solely with oleic acid and linked to the candidate gene *NAT10* (*putative nucleobase-ascorbate transporter 10*).

**Figure 6:**
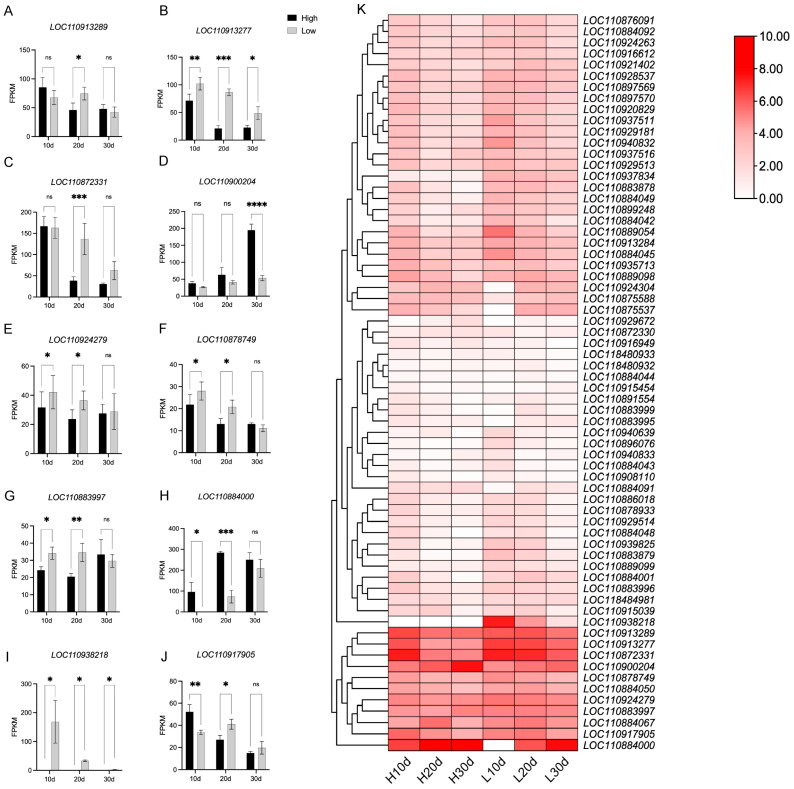
Expression patterns of candidate genes in sunflower inbred lines with different fatty acid contents. (**A**–**C**) Linoleic acid-related genes; (**D**–**F**) Palmitic acid-related genes; (**G**–**J**) Oil content-related genes; High, high-oleic acid variety; Low, low-oleic acid variety; (**K**) The heatmap is based on FPKM values from transcriptome data. (High (**H**), high-oleic acid variety; Low (**L**), low-oleic acid variety; ns, non-significant; *, ** and *** indicate 0.05, 0.01, and 0.001 levels of significance; 10d, 20d, 30d: 10, 20, and 30 days after pollination, respectively).

To assess the potential role of candidate genes in regulating quality-related traits, we analyzed their expression patterns using our previously published transcriptome data (NCBI: PRJNA1247595) [20] from sunflower seeds with varying fatty acid contents at 10, 20, and 30 days after pollination (DAP). 66 candidate genes showed expression during seed development (Figure 6). Within 30 DAP, 12 genes (*LOC110913289*, *LOC110913277*, *LOC110872331*, *LOC110924279*, *LOC110900204*, *LOC110878749*, *LOC110884000*, *LOC110883997*, *LOC110917905*, *LOC110938218*, *LOC110884050*, *LOC110884067*) exhibited significant differential expression between high-oleic and low-oleic varieties. The expression levels of *LOC110913277*, *LOC110924279*, and *LOC110872331* decreased with increasing DAP, whereas those of *LOC110884000* and *LOC110900204* increased. *LOC110884067* expression showed an initial increase followed by a decrease.

Functionally, these differentially expressed genes could be classified into several categories. *LOC110938218* (*HaDES8.11*) is a core enzyme in fatty acid desaturation. Genes such as *LOC110913277* (*RNC*, *Ribonuclease 3*) and *LOC110872331* (*RS25*, *ribosomal protein eS25*) are involved in RNA metabolism and translation, potentially influencing the overall protein synthesis capacity required for lipid biosynthesis. Others, including *LOC110884000* (*GLUB1*) and *LOC110883997* (*TM1L2*), are associated with storage protein accumulation and vesicular transport, processes linked to seed storage mobilization and oil body formation. Notably, *LOC110924279* (annotated as “--” in Table 2, suggesting an uncharacterized protein) and *LOC110917905* (*RA1L3*, *an RNA-binding protein*) may play roles in post-transcriptional or translational regulation. The coordinated expression changes of genes across these functional categories—from core metabolism to broader cellular processes—suggest a multi-layered regulatory network underpinning the variation in fatty acid content between high- and low-oleic acid sunflower inbred lines. These genes are hypothesized to be important regulators of fatty acid content-related traits in sunflower.

Notably, *LOC110938218* (*omega-6 fatty acid desaturase DES8.11*, *HaDES8.11*) was not expressed in high-oleic varieties but was significantly upregulated in low-oleic varieties. This observation aligns with the GWAS detection of this locus for oil content, suggesting that *HaDES8.11* may indirectly affect total oil accumulation through regulating fatty acid desaturation.

### 2.6. HaDES8.11 Increases Linoleic Acid Content in Arabidopsis Seeds

**Figure 7:**
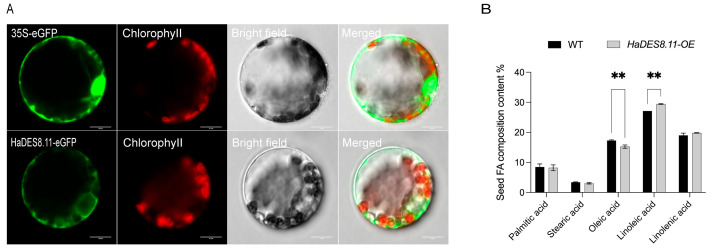
Subcellular localization of HaDES8.11-eGFP fusion protein in tobacco mesophyll protoplasts and seed fatty acid composition in Arabidopsis overexpression lines. (**A**) Subcellular localization of HaDES8.11 protein using HaDES8.11-eGFP in tobacco mesophyll protoplasts. Scale bar = 10 µm. (**B**) Oleic acid (C18:1) and linoleic acid (C18:2) content in seeds of wild-type and *HaDES8.11*-overexpressing Arabidopsis lines. Data are mean ± SD (n = 3 biological replicates). ** indicates significant difference at *p* < 0.01 (Student’s *t*-test).

Based on functional annotation, *LOC110938218* (*HaDES8.11*) was selected for functional validation. *HaDES8.11* was heterologously expressed in *Arabidopsis thaliana*, and fatty acid profiles were compared between overexpression lines and wild-type plants. Compared to wild-type, seeds from *HaDES8.11*-overexpressing Arabidopsis lines showed an 8.47% increase in linoleic acid content and a 12.11% decrease in oleic acid content. Subcellular localization analysis in tobacco (*Nicotiana benthamiana*) mesophyll protoplasts confirmed that the HaDES8.11-eGFP fusion protein specifically localizes to the endoplasmic reticulum (Figure 7).

## 3. Discussion

The integrated GWAS conducted across three environments provides a robust genetic mapping of five key quality traits in sunflower. A major strength of this study lies in the identification of stable SNP-trait associations detected consistently across multiple environments, which likely represent genetic loci with broad adaptability and are therefore highly valuable for marker-assisted breeding. This perspective is supported by advances in GWAS for oil crop quality traits. Specifically in sunflower, Gubaev et al. [17] defined a set of SNP markers for each cross that could be used for marker-assisted selection, confirming the practical utility of stable genetic loci in breeding programs. Furthermore, the discovery of several pleiotropic loci associated with multiple fatty acid traits (e.g., the SNP on chromosome 4 linked to oleic, linoleic, and stearic acids) underscores the interconnected genetic architecture governing oil composition. This suggests that targeted modification of key hub genes or genomic regions may simultaneously optimize multiple quality parameters.

Through multi-environment field trials and two-way ANOVA (Table S2), this study systematically evaluated the genotype-by-environment interaction patterns for quality traits in sunflower. The results demonstrated highly significant G×E interactions (*p* < 0.001) for all five traits, though the interaction intensities varied. Linoleic acid exhibited the largest environmental effect (F = 134.613) but a relatively smaller interaction effect (F = 3.433), suggesting its content is predominantly influenced by environment, and thus genetic improvement requires multi-year, multi-location evaluation under target environments. This pattern is consistent with previous multi-location trials in sunflower, which reported that linoleic acid content showed greater environmental plasticity compared to oil content [5,8]. Shah et al. [5] demonstrated through stability analysis that the oleic/linoleic acid ratio varied significantly across locations, emphasizing the necessity of multi-environment evaluation in high-oleic sunflower breeding.

Notably, we also identified trait-specific associations, offering precise targets for the independent adjustment of individual fatty acids. Among the candidate genes, core metabolic enzymes such as *KCS5* (*LOC110937516*) and *ω-6 desaturase* (*HaDES8.11*) were prominent, reaffirming the central role of canonical biosynthesis pathways. The combination of stable multi-environment signals, evidence of pleiotropy, and the pinpointing of both specific and core metabolic genes provides a comprehensive genetic blueprint for the systematic improvement of sunflower oil quality. The *KCS* family constitutes a key component of the fatty acid elongase complex. Previous studies in Arabidopsis have confirmed that *KCS5* can partially rescue the phenotype of fatty acid *elongase 1* (*FAE1*) mutants, clearly demonstrating its function in very-long-chain fatty acid metabolism [21,22]. In sunflower, the multi-trait association of *LOC110937516* suggests it may influence the balance between saturated and unsaturated fatty acid content by regulating the fatty acid elongation process [23]. This hypothesis is supported by functional characterization studies: González-Mellado et al. demonstrated that two sunflower *KCS* cDNAs, when expressed in yeast, altered the host’s fatty acid profile, proving that the encoded KCSs are functional and contribute to the presence of very-long-chain fatty acids in sunflower oil. These studies provide functional evidence for the conserved role of *KCS* genes in fatty acid elongation, supporting our findings [24]. Furthermore, comparative analysis of *KCS* genes across Asteraceae species has revealed conserved domains essential for fatty acid elongation, providing evolutionary evidence for their role in lipid metabolism [25,26].

The co-detection of *LOC110872472* (*G-type lectin S-receptor-like serine/threonine-protein kinase*) with oleic and linoleic acids expands our understanding of regulatory pathways for fatty acids. This class of genes is traditionally implicated in plant stress response and developmental regulation. In Arabidopsis, *G-type lectin S-receptor-like kinases* can activate immune signaling pathways through the FLS2-BAK1 complex [27]. Its association with fatty acid traits in our study suggests it may indirectly regulate oil metabolism through signal transduction pathways. On one hand, stress signals might be transmitted via this kinase, influencing the expression of oil synthesis-related genes to adapt to environmental changes. On the other hand, it might participate in intercellular signaling, coordinating oil accumulation in the achenes of the capitulum. This finding challenges the traditional view that fatty acid metabolism is regulated solely by core metabolic genes, revealing the potential role of signal transduction in oil synthesis. This perspective is consistent with recent findings from sunflower lipidomics studies. Chernova et al. [28] performed genotyping and lipid profiling of 601 cultivated sunflower lines and identified multiple genetic loci associated with fatty acid composition distributed across various chromosomes, involving genes beyond core metabolic enzymes. Furthermore, Gubaev et al. demonstrated through QTL mapping that while a single major-effect QTL governs high oleic acid content, its phenotypic expression varies significantly across genetic backgrounds, suggesting complex interactions between genotype and genetic context that imply a sophisticated regulatory network [17].

The association of palmitic acid content with *LOC110883878* (*myricetin 3-O-methyltransferase 3*) and *LOC110924851* (F-box/kelch-repeat protein) implicates secondary metabolism and protein degradation in its regulation. Myricetin 3-O-methyltransferase primarily catalyzes the methylation of flavonoid compounds. Flavonoids may indirectly regulate fatty acid metabolism by influencing metabolic flux, potentially competing for common acyl precursors and altering palmitic acid synthesis efficiency [29]. F-box/kelch-repeat proteins participate in the ubiquitin-26S proteasome pathway and could promote palmitic acid accumulation by degrading negative regulators of its synthesis [30]. While these hypotheses are consistent with our GWAS findings, functional validation—such as gene silencing or overexpression studies—will be necessary to establish causal relationships.

The association of stearic acid with *LOC110889054* (*inositol polyphosphate 5-phosphatase 9*, *IP5P9*) highlights the regulatory role of the phosphatidylinositol signaling pathway, which is important in plant lipid metabolism. This pathway can influence fatty acid synthesis and transport by modulating membrane phospholipid metabolism. As a key enzyme in this pathway, *LOC110889054* might alter the structure and function of cell membranes by regulating inositol phosphate levels, thereby affecting stearic acid synthesis and storage. This aligns with the theory proposed by Xing et al. [31]. on phospholipid signaling regulating lipid metabolism and provides a new direction for the genetic improvement of stearic acid content.

The association of linoleic acid with *LOC110918054* (*laccase-13*) and *LOC110886018* (*nucleobase-ascorbate transporter 10*, *NAT10*) reflects the influence of cell structure and substance transport on oil accumulation. Laccases are involved in cell wall lignification. The structural integrity of the cell wall might affect the storage space for oils within the seed; excessive lignification could compress the intercellular space available for oil storage, indirectly reducing linoleic acid content [32]. The nucleobase-ascorbate transporter maintains cellular redox homeostasis by regulating ascorbate transport. Ascorbate can protect the activity of linoleic acid synthesis-related enzymes by scavenging reactive oxygen species, reducing oxidative damage that might inhibit linoleic acid accumulation [33]. Together, these genes suggest that linoleic acid metabolism results from the coordinated action of cell structure, substance transport, and redox balance.

The repeated detection of *E3 ubiquitin ligases* (e.g., *LOC110937834*) and F-box proteins in this study underscores the central role of ubiquitination in fatty acid metabolism. The ubiquitin-26S proteasome pathway is a key mechanism for regulating protein stability in plants. In fatty acid metabolism, it could promote fatty acid accumulation by precisely degrading negative regulators (e.g., proteins that inhibit fatty acid metabolic enzymes), thereby releasing enzymatic activity [34]. Recent studies in cotton have demonstrated that the RING domain-containing E3 ubiquitin ligase *GhATL68b* regulates the stability of 2,4-dienoyl-CoA reductase, a rate-limiting enzyme in polyunsaturated fatty acid β-oxidation, through ubiquitination, thereby affecting fiber cell development and fatty acid composition. Knockout mutants of this gene showed significantly reduced polyunsaturated fatty acid content, and fiber growth defects could be fully rescued by supplementation with linolenic acid [35]. This finding directly confirms the molecular mechanism by which *E3 ubiquitin ligases* regulate fatty acid metabolism through the proteasome pathway. These studies provide important references for understanding the potential roles of ubiquitination-related genes identified in this study in sunflower fatty acid metabolism. However, direct evidence for their role in sunflower lipid metabolism remains to be established, and functional validation of these candidate genes is warranted.

Although our GWAS identified 154 candidate genes associated with the five quality traits, functional validation in this study focused on *HaDES8.11* (*LOC110938218*) for several strategic reasons. First, this gene was prioritized based on its well-established biochemical role in fatty acid desaturation—converting oleic acid (18:1) to linoleic acid (18:2)—which is a critical step determining the final fatty acid composition in oilseed crops [36]. Second, transcriptome analysis revealed that *HaDES8.11* exhibited the most pronounced differential expression between high- and low-oleic acid varieties (Figure 6), suggesting its potential as a master regulator of the oleic/linoleic acid balance. Third, the significant SNP associated with this gene was consistently detected across multiple environments and in the BLUE analysis (Table 2), indicating its stable contribution to oil content variation. Functional validation in *Arabidopsis thaliana* confirmed that *HaDES8.11* encodes a functional ω-6 desaturase, as its overexpression significantly increased linoleic acid (18:2) content while decreasing oleic acid (18:1) content, consistent with the known role of FAD2-family enzymes in converting oleic acid to linoleic acid. The apparent discrepancy between the GWAS association (oil content) and the functional validation (fatty acid composition) can be explained by several plausible and interconnected reasons. First, a strong genetic correlation often exists between oil content and fatty acid composition in oilseeds; correlation analysis (Figure 2) revealed a very strong negative correlation between oleic and linoleic acid content (r = −0.89, *p* < 0.001), while oil content showed a positive trend with oleic acid (r = 0.18, *p* = 0.12) and no significant correlation with linoleic acid (r = 0.09, *p* = 0.35). This indicates that when *HaDES8.11* activity alters the flux from oleic to linoleic acid, although the direct effect is on fatty acid composition, this change may indirectly affect total oil accumulation through metabolic reallocation [36,37]. Thus, the GWAS signal for oil content could have been captured due to this underlying physiological linkage. Second, in the natural population used for GWAS, the identified SNP might be in linkage disequilibrium (LD) with other, uncharacterized causal variant(s) that directly regulate oil content, while the *HaDES8.11* allele itself primarily governs fatty acid profile. This highlights a common scenario in GWAS where the statistically associated marker pinpoints a genomic region harboring multiple genes with related but distinct functions.

For the remaining 153 candidate genes, future validation efforts will be prioritized according to a multi-tiered strategy. High-priority candidates include genes with known or predicted functions in lipid metabolism (e.g., *KCS5*), genes co-localized with multi-trait associated SNPs, and genes showing expression correlations with fatty acid content during seed development. These will be subjected to reverse genetic approaches such as virus-induced gene silencing (VIGS) in sunflower or heterologous overexpression in Arabidopsis or tobacco. Medium-priority candidates will first be evaluated through expression profiling, followed by functional validation of the most promising candidates. This systematic validation pipeline will progressively elucidate the functional roles of the candidate genes identified in this study and their contributions to sunflower oil quality.

## 4. Materials and Methods

### 4.1. Plant Materials and Cultivation

The plant materials comprised 203 oilseed sunflower inbred lines, preserved and provided by the Oilseed Crop Germplasm Innovation Team of Inner Mongolia Agricultural University, the Inner Mongolia Academy of Agricultural & Animal Husbandry Sciences, and the Bayannur Academy of Agricultural & Animal Husbandry Sciences. This collection included 150 domestic accessions and 53 introduced from Russia, the USA, Pakistan, Canada, and other countries.

Field trials were conducted in 2022 at three locations in Hohhot, Inner Mongolia, China: (1) the experimental base of the Inner Mongolia Academy of Agricultural & Animal Husbandry Sciences (NKY) in Yuquan District (E111°67′, N40°75′, loam soil, active accumulated temperature (AAT) 2400 °C, rainfall 450 mm, soil pH 7.6); (2) Qigetai Village (QGT) in Saihan District (E111°74′, N40°69′, loam soil, AAT 2300 °C, rainfall 480 mm, soil pH 6.5); and (3) the Science and Technology Park of Inner Mongolia Agricultural University in Hailiutu (TZQ), Tumd Left Banner (E111°28′, N40°38′, loam soil, AAT 2350 °C, rainfall 400 mm, soil pH 8.6). A completely randomized block design with three replications was used. Plots consisted of three rows, each 6 m long, with row spacing of 0.7 m and plant spacing of 0.3 m. Manual dibbling was used for sowing. Standard field management practices, including timely irrigation and weeding, were followed.

### 4.2. Phenotypic Evaluation and Data Analysis

Oil content and fatty acid composition (linoleic acid, oleic acid, stearic acid, palmitic acid) were determined for mature seeds using a near-infrared analyzer (Perten DA7250, Stockholm, Sweden). NIR model calibration statistics are oleic acid (R: 0.82, RMSEC: 1.5), linoleic acid (R: 0.89, RMSEC: 0.95), stearic acid (R: 0.87, RMSEC: 0.43), palmitic acid (R: 0.82, RMSEC: 0.22), oil content (R: 0.98, RMSEC: 0.39). One-way ANOVA, two-way ANOVA, broad-sense heritability calculation, and other statistical analyses were performed using R (v4.1.0) and Prism 10.

### 4.3. Whole-Genome Resequencing, SNP Calling, and Annotation

Genomic DNA was extracted from young leaves using a plant genomic DNA extraction kit (Tiangen DP338). DNA libraries were constructed and sequenced on the DNBSEQ-T7 platform. Raw sequencing reads were processed to remove adapter sequences and low-quality bases using fastp (v0.23.2) [38]. Clean reads were aligned to the sunflower reference genome (HanXRQr2.0, GenBank assembly GCA_002127325.2) using BWA (v0.7.8). SNP calling was performed using SAMTOOLS (v1.3) [18]. The resulting raw SNPs were filtered using VCFtools (v0.1.16) [34] with the following criteria: depth ≥ 4, missing rate < 0.2, and minor allele frequency (MAF) > 0.01, resulting in 1,334,041 high-quality SNPs for subsequent analysis. SNP annotation was performed using ANNOVAR (v2013-05-20) [39].

### 4.4. Population Structure, Phylogenetic Analysis, and Linkage Disequilibrium

To account for population stratification in GWAS, population structure was analyzed using ADMIXTURE (v1.23) [40] with K values ranging from 2 to 8. The optimal K was determined by the lowest cross-validation error. A neighbor-joining phylogenetic tree was constructed using TreeBeST (v1.9.2) based on a pairwise distance matrix calculated from the SNP data. Principal component analysis (PCA) was conducted using GCTA (v1.24.2) [41]. Linkage disequilibrium (LD) decay was analyzed using PopLDdecay (v3.40) [42] by calculating the squared correlation coefficient (r^2^) between pairwise SNPs within a 500 kb window. The genome-wide LD decay distance was estimated as the physical distance at which the average r^2^ dropped to half its maximum value.

### 4.5. Genome-Wide Association Study (GWAS)

GWAS was performed separately for each trait using the filtered SNP dataset and phenotypic data (individual environment means and BLUE values). The mixed linear model (MLM) accounting for population structure (Q) and kinship (K) was implemented in GAPIT (v3) [43] and/or GEMMA (v0.98.1) [44]. The model was: y = Xα + Qβ + Kμ + e, where y is the phenotype vector, X is the genotype matrix, α is the SNP effect, Q is the population structure matrix (from PCA), β is the coefficient for population structure, K is the kinship matrix, μ is the random polygenic effect, and e is the residual error. A significance threshold was set at −log_10_(*p*) = 5.0. The significance threshold was set at −log_10_(*p*) = 5.0. (i.e., *p* = 1 × 10^−5^). This threshold was chosen based on empirical considerations to balance false positive control and detection power. Given the effective number of SNPs in this study (1,334,041), the strict Bonferroni-corrected threshold (−log_10_(0.05/1,334,041) ≈ 7.43) would be overly conservative and could lead to the omission of true associations. Therefore, the widely used empirical threshold of −log_10_(*p*)= 5 in genetic studies was adopted [9]. Multiple testing correction was applied separately for each environment and the BLUE analysis. SNPs surpassing this threshold were considered significantly associated with the trait.

### 4.6. Candidate Gene Prediction and Expression Analysis

Candidate genes were identified within a 20 kb region upstream and downstream of each significant SNP based on the LD decay distance. Gene annotations were retrieved from the sunflower reference genome (HanXRQr2.0). Publicly available transcriptome data (NCBI SRA: PRJNA1247595) from developing sunflower seeds were analyzed. This dataset contains transcriptome sequencing data from seeds of high-oleic acid inbred line (227) and low-oleic acid inbred line (228) at 10, 20, and 30 days after pollination. Raw sequencing reads were quality-controlled using fastp (v0.23.2) to remove adapter sequences and low-quality bases. Clean reads were aligned to the sunflower reference genome using HISAT2 (v2.2.1). Transcript assembly was performed with StringTie (v2.2.1), and FPKM (fragments per kilobase per million) values for each gene were calculated using the ballgown R package. Differentially expressed genes were identified using the criteria of |log_2_(Fold Change)| ≥ 1 and FDR < 0.05. Heatmaps of candidate gene expression were generated using TBtools (v2.4.20) software [45].

### 4.7. Vector Construction and Plant Transformation

The full-length coding sequence (CDS) of *HaDES8.11* (*LOC110938218*) was amplified from sunflower cDNA using gene-specific primers (Supplementary Table S4) and cloned into the pBWA(V)BS-linker-egfp vector (provided by Wuhan Bioyuan Biotechnology Co., Ltd., Wuhan, China) via homologous recombination, generating a 35S:HaDES8.11-eGFP fusion construct. Arabidopsis was transformed using the dipping method mediated by *Agrobacterium* [46]. Homozygous T3 transgenic Arabidopsis seeds were used for fatty acid analysis.

### 4.8. Subcellular Localization

The 35S:HaDES8.11-eGFP construct and were transfected into tobacco (Nicotiana benthamiana) mesophyll protoplasts prepared as described previously [47]. After 16–20 h of incubation in the dark at 22 °C, fluorescence signals were observed using a confocal laser scanning microscope (Nikon AX, Kyoto, Japan). GFP was excited at 488 nm, and Chloroplast fluorescence signal excitation at 640 nm.

### 4.9. Fatty Acid Analysis of Arabidopsis Seeds

FAs were extracted from mature seeds using the gas chromatography (GC) FA methyl ester method, following Chen et al. [48]. Various FA species were measured with GC (Shimadzu-2014, Kyoto, Japan).

## 5. Conclusions

This study presents an integrated genetic analysis of quality traits in sunflower inbred lines by combining multi-environment GWAS with transcriptomic and functional validation. We systematically dissected the genetic architecture underlying oil content and fatty acid composition, identifying a set of stable SNP markers and candidate genes. The discovery of both pleiotropic loci (influencing multiple traits) and trait-specific associations provides a nuanced genetic blueprint for quality improvement. We identified 103 significant SNPs and 154 candidate genes associated with oil content, oleic acid, linoleic acid, palmitic acid, and stearic acid. Preliminary functional characterization of the *ω-6 fatty acid desaturase gene HaDES8.11* (*LOC110938218*) confirmed its role in modulating the oleic/linoleic acid ratio, with overexpression in Arabidopsis increasing linoleic acid and decreasing oleic acid content, and verified its endoplasmic reticulum localization. These findings provide valuable genomic resources and candidate genes for marker-assisted selection and genetic engineering aimed at improving oil quality traits in sunflower breeding programs.

## Figures and Tables

**Table 1:** Statistical analysis of sunflower quality traits.

Trait	Environment	Min (%)	Max (%)	Mean (%)	SD (%)	CV (%)	H^2^ (%)
Linoleic acid(%)	NKY	63.13	69.37	65.93	1.09	1.65	48.25
	QGT	62.59	69.67	65.56	0.97	1.48	
	TZQ	61.78	70.63	65.55	1.03	1.58	
Oleic acid(%)	NKY	18.77	27.68	23.12	1.58	6.84	49.45
	QGT	18.54	28.20	23.47	1.55	6.62	
	TZQ	17.75	28.44	23.39	1.55	6.64	
Stearic acid(%)	NKY	0.19	8.58	4.95	1.41	28.45	64.12
	QGT	0.24	8.56	4.77	1.38	28.82	
	TZQ	0.13	9.22	4.82	1.36	28.13	
Palmitic acid(%)	NKY	5.92	6.98	6.36	0.21	3.35	56.13
	QGT	5.82	7.07	6.29	0.21	3.34	
	TZQ	5.78	7.17	6.30	0.21	3.29	
Oil content(%)	NKY	27.08	49.05	37.62	3.88	10.31	70.78
	QGT	28.98	49.70	38.62	3.80	9.84	
	TZQ	28.40	48.65	38.36	3.64	9.49	

Note: SD, standard deviation; CV, coefficient of variation (%); H^2^, broad-sense heritability; Mean, per-environment mean; NKY was planted in 2022 at the Experimental Base of the Institute of Agricultural Sciences of Inner Mongolia Autonomous Region, Yuquan District, Hohhot; QGT was planted in 2022 at Qigetai Village, Saihan District, Hohhot; TZQ was planted in 2022 at the Hailitu Campus of Inner Mongolia Agricultural University, Tumed Left Banner, Hohhot.

**Table 2:** The potential candidate genes identified corresponding to the significant SNP associated with 5 quality-related traits in the population.

Traits	SNP Position (bp)	Chr	Gene ID	Position (bp)	Distance (bp)	Environment	PVE (%)	Gene Annotation
Stearic acid, Linoleic acid, Oleic acid	6,375,699	3	*LOC110928537*	6,398,709–6,403,894	−17,825	QGT, TZQ, BLUE (Sa) QGT, TZQ, BLUE (La) NKY, QGT, TZQ, BLUE (Oa)	9.16	NNJA2 Ninja-family protein 2
Stearic acid, Linoleic acid, Oleic acid	133,076,174	3	*LOC110929672*	133,063,851–133,070,872	12,323	NYK, QGT, BLUE (Sa) NKY, QGT, BLUE (La) NKY, QGT, BLUE (Oa)	8.45	-- --
Stearic acid, Linoleic acid, Oleic acid	185,951,879	4	*LOC110937516*	185,972,636–185,974,662	−18,731	NYK, QGT, BLUE (Sa) NKY, QGT, TZQ, BLUE (La) NKY, QGT, BLUE (Oa)	13.24	KCS5 3-ketoacyl-CoA synthase 5
Stearic acid, Linoleic acid, Oleic acid	185,951,879	4	*LOC110937511*	185,939,063–185,943,747	12,816	NYK, QGT, BLUE (Sa) NKY, QGT, TZQ, BLUE (La) NKY, QGT, BLUE (Oa)	13.24	IQD20 Protein IQ-DOMAIN 20
Oleic acid	146,673,044	10	*LOC110886018*	146,670,623–146,674,866	2421	QGT, BLUE	9.15	NAT10 Putative nucleobase-ascorbate transporter 10
Stearic acid, Oleic acid	466,927	11	*LOC110889054*	476,710–488,913	0	NYK, QGT, TZQ, BLUE (Sa) NYK, QGT, TZQ, BLUE (Oa)	9.48	IP5P9
Linoleic acid	22,847,761	2	*LOC110913289*	22,824,514–22,827,480	20,281	QGT, BLUE	8.34	SEM12 Protein DSS1
Linoleic acid	22,847,761	2	*LOC110913284*	22,830,564–22,834,169	13,592	QGT, BLUE	8.34	-- --
Linoleic acid	22,847,761	2	*LOC110913277*	22,853,370–22,855,128	−5609	QGT, BLUE	8.34	RNC Ribonuclease 3
Linoleic acid	54,329,976	3	*LOC110929181*	54,342,214–54,346,799	−12,238	QGT, BLUE	9.46	VDAC1 Mitochondrial outer membrane protein porin 1
Linoleic acid	111,419,824	3	*LOC110929514*	111,384,465–111,402,514	17,310	QGT, BLUE	8.67	TOR Serine/threonine-protein kinase TOR
Linoleic acid	111,419,824	3	*LOC110929513*	111,406,443–111,406,902	12,922	QGT, BLUE	8.67	DPH3 Diphthamide biosynthesis protein 3
Linoleic acid	11,122,991	4	*LOC110935713*	11,125,931–11,130,693	−2940	QGT, BLUE (La) NYK, QGT, BLUE (Sa)	12.67	-- --
Linoleic acid, Stearic acid	199,014,203	4	*LOC110937834*	198,991,368–198,997,034	17,169	QGT, BLUE	8.65	RING1 E3
Linoleic acid	6,929,244	5	*LOC110939825*	6,941,239–6,944,498	−11,995	QGT, BLUE	8.24	KGUA Guanylate kinase
Linoleic acid	29,260,500	5	*LOC110940639*	29,265,531–29,269,300	−5031	QGT, TZQ, BLUE	6.48	Y1534 Probable LRR receptor-like
Linoleic acid	98,465,304	5	*LOC110940833*	98,468,630–98,471,613	−3326	QGT, TZQ, BLUE	9.13	SLAH4 S-type anion channel SLAH4
Linoleic acid	98,465,304	5	*LOC110940832*	98,475,645–98,479,065	−10,341	QGT, TZQ, BLUE	9.13	-- --
Linoleic acid	21,589,268	8	*LOC110872331*	21,625,078–21,626,902	−35,810	NKY, QGT, BLUE	8.17	RS25 Small ribosomal subunit protein eS25
Linoleic acid	21,589,268	8	*LOC110872330*	21,628,888–21,631,133	−39,620	NKY, QGT, BLUE	8.17	LRK91 L-type lectin-domain containing receptor kinase IX.1
Linoleic acid	14,173,953	14	*LOC110908110*	14,154,205–14,158,357	15,596	TZQ, BLUE	9.31	PPR45 Pentatricopeptide repeat-containing protein
Linoleic acid	21,952,638	16	*LOC110916612*	21,940,895–21,942,297	10,341	NKY, QGT, TZQ, BLUE	7.16	AIPP1 ASI1-immunoprecipitated protein 1
Palmitic acid	55,590,240	1	*LOC110878749*	55,608,429–55,612,095	3667	TZQ, BLUE	8.27	AP1B1 AP-1 complex subunit beta-1
Palmitic acid, Oleic acid	94,520,495	1	*LOC110876091*	94,501,658–94,505,359	18,837	QGT, TZQ, BLUE (Pc) TZQ, BLUE (Oa)	9.26	CC174 Coiled-coil domain-containing protein 174
Palmitic acid	151,232,052	2	*LOC110924304*	151,235,213–151,249,821	14,609	QGT, BLUE	6.18	CSLA1 Probable glucomannan 4
Palmitic acid	151,232,052	2	*LOC110924279*	151,215,514–151,218,182	2669	QGT, BLUE	6.18	-- --
Palmitic acid	151,232,052	2	*LOC110924263*	151,209,797–151,211,508	1712	QGT, BLUE	6.18	TAF8 Transcription initiation factor TFIID subunit 8
Palmitic acid	42,766,787	9	*LOC110875588*	42,784,492–42,784,965	474	NKY, TZQ, BLUE	5.12	ATL51 RING-H2
Palmitic acid	6,871,947	10	*LOC110883878*	6,865,269–6,873,805	8537	NKY, TZQ, BLUE	7.13	MOMT3 Myricetin 3-O-methyltransferase 3
Palmitic acid	6,723,037	10	*LOC110883879*	6,739,640–6,743,919	4280	NKY, TZQ, BLUE	9.38	Y5360 Putative BTB/POZ domain-containing protein
Palmitic acid	26,755,804	13	*LOC110897570*	26,753,878–26,757,281	3404	TZQ, BLUE	9.79	NSE4 Non-structural maintenance of chromosome element 4
Palmitic acid	26,755,804	13	*LOC110897569*	26,732,177–26,737,833	5657	TZQ, BLUE	9.79	C3H36 Zinc finger CCCH domain-containing protein 36
Palmitic acid	123,353,062	13	*LOC110899248*	123,353,941–123,365,809	11,869	QGT, TZQ, BLUE	6.37	RGAP6 Rho GTPase-activating protein 6
Palmitic acid	164,943,371	13	*LOC110900204*	164,953,671–164,958,478	4808	QGT, TZQ, BLUE	10.42	CYT3 Cysteine proteinase inhibitor 3
Palmitic acid, Oil content	105,985,397	16	*LOC110920829*	105,975,869–105,992,994	17,126	NKY, QGT, BLUE (Pc) NKY, QGT, TZQ, BLUE (Oc)	7.64	MAST4 Microtubule-associated serine/threonine-protein kinase 4
Palmitic acid	13,629,538	16	*LOC110916949*	13,620,569–13,623,616	3048	TZQ, BLUE	6.48	HERK Receptor-like protein kinase HERK 1
Stearic acid	26,775,458	8	*LOC118480933*	26,767,890–26,769,769	5689	NYK, QGT, BLUE	9.47	-- --
Stearic acid	26,775,458	8	*LOC118480932*	26,766,177–26,767,142	8316	NYK, QGT, BLUE	9.47	-- --
Stearic acid	158,033,685	12	*LOC110896076*	158,028,236–158,037,793	0	NYK, BLUE	11.27	KNAP2 Homeobox protein knotted-1-like 2
Stearic acid	201,138,278	16	*LOC110915454*	201,128,981–201,144,251	0	NYK, QGT, TZQ, BLUE	7.96	MA2B1 Lysosomal alpha-mannosidase
Stearic acid	31,077,648	17	*LOC110891554*	31,079,163–31,079,816	−1515	NYK, QGT, TZQ, BLUE	10.16	-- --
Stearic acid	31,077,648	17	*LOC110889099*	31,083,590–31,092,385	−5942	NYK, QGT, TZQ, BLUE	10.16	DTX44 Protein DETOXIFICATION 44
Stearic acid	31,077,648	17	*LOC110889098*	31,058,210–31,060,394	17,254	NYK, QGT, TZQ, BLUE	10.16	-- --
Oil content	206,672,182	4	*LOC110938218*	206,690,013–206,691,525	−17,831	NKY, QGT, BLUE	14.27	Omega-6 fatty acid desaturase, endoplasmic reticulum isozyme 1
Oil content	107,198,011	9	*LOC110878933*	107,190,260–107,194,387	3624	NKY, QGT, TZQ, BLUE	6.57	BZP02 Basic leucine zipper 2
Oil content	81,488,924	9	*LOC110875537*	81,524,552–81,551,420	−35,628	NKY, QGT, BLUE	7.85	LHTL2 Lysine histidine transporter-like 2
Oil content	13,962,168	10	*LOC110884092*	14,484,368–14,486,031	−522,200	NKY, QGT, TZQ, BLUE	7.96	-- --
Oil content	13,962,168	10	*LOC110884091*	14,424,352–14,430,527	−462,184	NKY, QGT, TZQ, BLUE	7.96	-- --
Oil content	13,962,168	10	*LOC110884050*	13,873,113–13,878,263	83,905	NKY, QGT, TZQ, BLUE	7.96	KAT2A Histone acetyltransferase KAT2A
Oil content	13,962,168	10	*LOC110884067*	14,011,954–14,014,319	−49,786	NKY, QGT, TZQ, BLUE	7.96	-- --
Oil content	13,962,168	10	*LOC110884049*	13,863,354–13,864,605	97,563	NKY, QGT, TZQ, BLUE	7.96	AUX28 Auxin-induced protein AUX28
Oil content	13,962,168	10	*LOC110884048*	13,792,898–13,798,086	164,082	NKY, QGT, TZQ, BLUE	7.96	FBL17 F-box/LRR-repeat protein 17
Oil content	13,962,168	10	*LOC110884045*	13,754,799–13,762,895	199,273	NKY, QGT, TZQ, BLUE	7.96	GSHR Glutathione reductase
Oil content	13,962,168	10	*LOC110884044*	13,735,026–13,736,830	225,338	NKY, QGT, TZQ, BLUE	7.96	PSBW Photosystem II reaction center W protein, chloroplastic
Oil content	13,962,168	10	*LOC110884043*	13,728,567–13,732,661	229,507	NKY, QGT, TZQ, BLUE	7.96	MRS2B Magnesium transporter MRS2
Oil content	13,962,168	10	*LOC110884042*	13,737,841–13,742,237	219,931	NKY, QGT, TZQ, BLUE	7.96	GAUT8 Galacturonosyltransferase 8
Oil content	12,294,531	10	*LOC110883999*	11,935,278–11,937,019	357,512	NKY, QGT, TZQ, BLUE	9.63	SKI25 F-box/kelch-repeat protein
Oil content	12,294,531	10	*LOC110884001*	11,991,288–11,992,831	301,700	NKY, QGT, BLUE	9.63	-- --
Oil content	12,294,531	10	*LOC110884000*	11,968,210–11,970,738	323,793	NKY, QGT, BLUE	9.63	GLUB1 Glutelin type-B 1
Oil content	12,294,531	10	*LOC110883997*	11,920,108–11,925,055	369,476	NKY, QGT, TZQ, BLUE	9.63	TM1L2 TOM1-like protein 2
Oil content	12,294,531	10	*LOC110883996*	11,877,363–11,884,214	410,317	NKY, QGT, BLUE	9.63	PUB11 U-box domain-containing protein 11
Oil content	12,294,531	10	*LOC110883995*	11,859,248–11,862,853	431,678	NKY, QGT, BLUE	9.63	NAC76 NAC domain-containing protein 76
Oil content	96,987,192	12	*LOC118484981*	96,996,091–97,001,443	−8899	NKY, QGT, TZQ, BLUE	7.82	LAC10 Laccase-10
Oil content	148,400,476	16	*LOC110917905*	148,401,803–148,405,349	−1327	NKY, QGT, TZQ, BLUE	6.15	RA1L3 Heterogeneous nuclear ribonucleoprotein A1-like 3
Oil content	48,838,013	16	*LOC110915039*	48,816,969–48,821,470	16,543	QGT, TZQ, BLUE	7.66	Y2685 Uncharacterized transporter
Oil content	77,590,944	17	*LOC110921402*	77,579,097–77,583,501	7443	QGT, TZQ, BLUE	6.93	COL4 Zinc finger protein CONSTANS-LIKE 4

Note: The genes detected for expression in GWAS and RNA data are shown in the table, while the remaining candidate genes are listed in Table S3.

## Data Availability

The original contributions presented in this study are included in the article/Supplementary Material. Further inquiries can be directed to the corresponding author.

## Supplementary Materials

The following supporting information can be downloaded at: https://www.mdpi.com/article/10.3390/plants15070999/s1, Table S1: Various quality-related traits of 203 sunflower inbred lines in 3 environments; Table S2: ANOVA of the quality-related traits for the 203 sunflower inbred lines; Table S3: The potential candidate genes identified corresponding to the significant SNP associated with 5 quality-related traits in the population; Table S4: Primers sequences for *HaDES8.11* (*LOC110938218*) amplification.

## Abbreviations

The following abbreviations are used in this manuscript:

GWAS	Genome-wide association study
SD	Standard deviation
CV	Coefficient of variation (%)
H^2^	Broad-sense heritability
BLUE	Best linear unbiased estimate
SNP	Single-nucleotide polymorphism
Q-Q	Quantile–quantile
PVE	Phenotypic variation explained
DAP	Days after pollination
